# Ocozocoautla de Espinosa Virus and Hemorrhagic Fever, Mexico

**DOI:** 10.3201/eid1803.111602

**Published:** 2012-03

**Authors:** Maria N.B. Cajimat, Mary Louise Milazzo, Robert D. Bradley, Charles F. Fulhorst

**Affiliations:** University of Texas Medical Branch, Galveston, Texas, USA (M.N.B. Cajimat, M.L. Milazzo, C.F. Fulhorst);; Texas Tech University, Lubbock, Texas, USA (R.D. Bradley)

**Keywords:** Arenaviridae, arenavirus, Tacaribe serocomplex, hemorrhagic fever, viruses, Mexico, Ocozocoautla de Espinosa virus, deer mice, Peromyscus mexicanus

## Abstract

Arenavirus RNA was isolated from Mexican deer mice (*Peromyscus mexicanus*) captured near the site of a 1967 epidemic of hemorrhagic fever in southern Mexico. Analyses of nucleotide and amino acid sequence data indicated that the deer mice were infected with a novel Tacaribe serocomplex virus (proposed name Ocozocoautla de Espinosa virus), which is phylogenetically closely related to Tacaribe serocomplex viruses that cause hemorrhagic fever in humans in South America.

## Abstract 2

This novel virus is related to arenaviruses that cause hemorrhagic fever.

Tacaribe serocomplex viruses (family *Arenaviridae*, genus *Arenavirus*) comprise *Bear Canyon virus*, *Tamiami virus*, and *Whitewater Arroyo virus* in the United States; *Tacaribe virus* (TCRV) on Trinidad; *Chaparé virus* (CHPV) and *Machupo virus* (MACV) in Bolivia; *Guanarito virus* (GTOV) in Venezuela; *Junín virus* (JUNV) in Argentina; *Sabiá virus* (SABV) in Brazil; and 9 other species (*1*). Provisional species in the Tacaribe serocomplex include Big Brushy Tank virus, Catarina virus, Skinner Tank virus, and Tonto Creek virus in the United States (*2**–**4*), and Real de Catorce virus in Mexico (*5*).

Five members of the Tacaribe serocomplex (CHPV, GTOV, JUNV, MACV, and SABV) cause hemorrhagic fever in humans (*6**,**7*). Diseases caused by these viruses are zoonoses. Specific members of the rodent family Cricetidae (*8*) are the principal hosts of the Tacaribe serocomplex viruses for which natural host relationships have been well characterized. For example, the short-tailed cane mouse (*Zygodontomys brevicauda*) in western Venezuela is the principal host of GTOV (*9**,**10*), and the drylands vesper mouse (*Calomys musculinus*) in central Argentina is the principal host of JUNV (*11*).

The history of human disease in North America includes large epidemics of highly lethal hemorrhagic fever during 1545–1815 in Mexico (*12*). These epidemics primarily affected native inhabitants of the highlands. Medical historians theorized that the hemorrhagic fever was caused by Tacaribe serocomplex virus(es) or other viruses associated with rodents native to Mexico (*13*).

A recently published study reported antibody against a Tacaribe serocomplex virus in 3 (25.0%) of 12 Mexican deer mice (*Peromyscus mexicanus*) and 0 of 29 other cricetid rodents captured in the municipality of Ocozocoautla de Espinosa, State of Chiapas, Mexico (*14*). Analyses of serologic data suggested that the 3 antibody-positive deer mice were infected with an arenavirus that is antigenically more closely related to the South American hemorrhagic fever arenaviruses than to other North American Tacaribe serocomplex viruses. The objective of this study was to determine the identity of the Tacaribe serocomplex virus associated with *P. mexicanus* deer mice in western Chiapas.

## Materials and Methods

**Figure 1:**
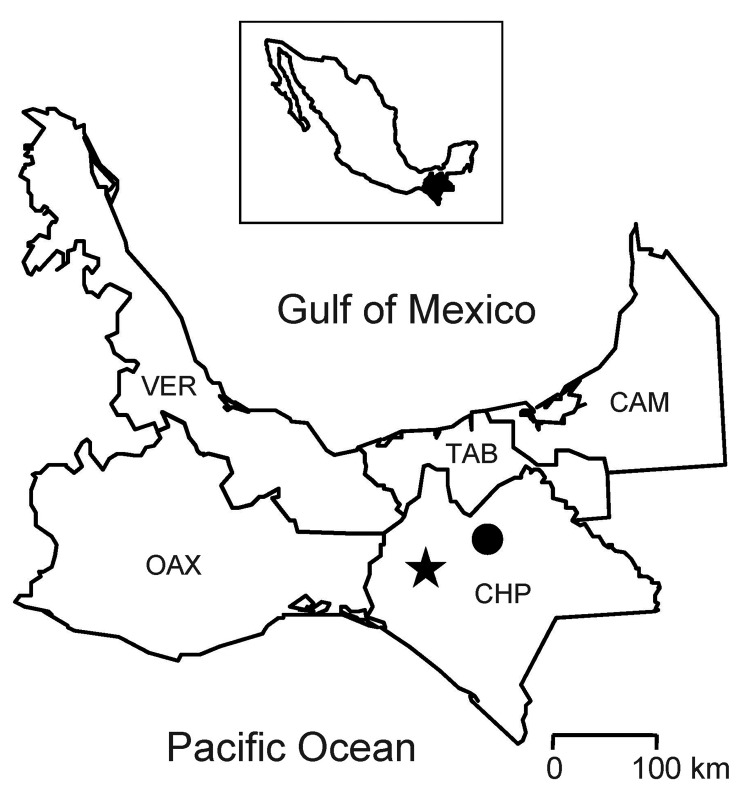
Chiapas (CHP) and surrounding states in southern Mexico. The star indicates where the rodents in this study were captured; the solid circle indicates the location of the hospital that provided care for the persons affected by hemorrhagic fever in the 1967 epidemic (*17*). Inset shows the location of CHP in Mexico. CAM, Campeche; OAX, Oaxaca; TAB, Tabasco; VER, Veracruz.

Kidney samples from the 3 antibody-positive Mexican deer mice, Mexican deer mouse TK93314 (*15*) and 8 other antibody-negative Mexican deer mice, 18 southern pygmy mice (*Baiomys musculus*), and 11 Jaliscan cotton rats (*Sigmodon mascotensis*) were tested for arenavirus by cultivation in monolayers of Vero E6 cells (*16*). The 3 antibody-positive deer mice (TK93319, TK93321, TK93325) and 38 antibody-negative rodents were captured on July 16, 2000, at a locality (Universal Transverse Mercator coordinates 15–451772E, 1864243N; elevation 1,021 m) in the municipality of Ocozocoautla de Espinosa (Figure 1).

Kidney samples from the 3 antibody-positive animals also were tested for arenavirus nucleocapsid (N) protein gene RNA. First-strand cDNA was synthesized by using SuperScript III Reverse Transcriptase (Invitrogen Corp., Carlsbad, CA, USA) and oligont 19C-cons (*18*). The first-round PCR used Master*Taq* Kit (5 PRIME, Inc., Gaithersburg, MD, USA) and 19C-cons and either AVNP42 (5′-GCCGCGGACTGGGAGGGCA-3′) or AVNP122 (5′-GCCGCGGACTGGGGAGGCACTG-3′). The second-round (heminested) PCR used Master*Taq* Kit and either AVNP42 and oligont 1010C (*19*), AVNP122 and 1010C, or AVNP115 (5′-CCAATATAAGGCCAACCATCG-3′) and AVNP149 (5′-CGCACAGTGGATCCTAGGCATAGTGTC-3′). The nucleotide sequence of a 3,380-nt fragment of the small genomic segment of arenavirus AV B1030026 (GenBank accession no. JN897398) was then determined from the first-strand cDNA from antibody-positive deer mouse TK93325 by using a series of 3 heminested PCRs. The 3,380-nt fragment extended from within the 5′ noncoding region, through the glycoprotein precursor (GPC) gene, intergenic region, and N protein gene, and into the 3′ noncoding region.

**Figure 2:**
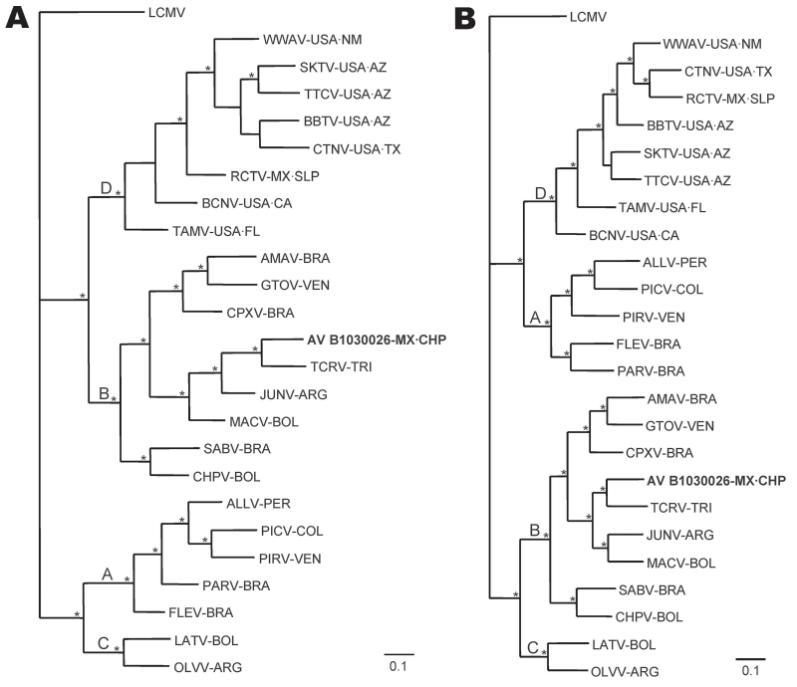
Phylogenetic relationships among Tacaribe serocomplex viruses from the United States, Mexico, and South America, as determined on the basis of Bayesian analyses of A) full-length glycoprotein precursor gene sequences and B) full-length nucleocapsid protein gene sequences. Arenavirus AV B1030026 is shown in **boldface**. Scale bars indicate nucleotide substitutions per site. Probability values in support of the clades were calculated a posteriori, clades with probability values >0.95 were considered supported by the data (*20*), and asterisks at nodes indicate that clades were supported by the data. LCMV, lymphocytic choriomeningitis virus, strain WE (GenBank accession no. M22138); WWAV, Whitewater Arroyo virus, AV 9310135 (AF228063); SKTV, Skinner Tank virus, AV D1000090 (EU123328); TTCV, Tonto Creek virus, AV D0150144 (EF619033); BBTV, Big Brushy Tank virus, AV D0390174 (EF619035); CTNV, Catarina virus, AV A0400135 (DQ865244); RCTV, Real de Catorce virus, AV H0030026 (GQ903697); BCNV, Bear Canyon virus, AV A0070039 (AY924391); TAMV, Tamiami virus, W 10777 (AF512828); AMAV, Amaparí virus, BeAn 70563 (AF512834); GTOV, Guanarito virus, INH-95551 (AY129247); CPXV, Cupixi virus, BeAn 119303 (AF512832); arenavirus AV B1030026 (JN897398); TCRV, Tacaribe virus, TRVL 11573 (M20304); JUNV, Junín virus, XJ13 (AY358023); MACV, Machupo virus, Carvallo (AY129248); SABV, Sabiá virus, SPH 114202 (U41071); CHPV, Chaparé virus, 200001071 (EU260463); ALLV, Allpahuayo virus CLHP-2472 (AY012687); PICV, Pichindé virus, Co An 3739 (K02734); PIRV, Pirital virus, VAV-488 (AF485262); PARV, Paraná virus, 12056 (AF485261); FLEV, Flexal virus, BeAn 293022 (AF512831); LATV, Latino virus, MARU 10924 (AF512830); OLVV, Oliveros virus, 3229–1 (U34248). Locations: NM, New Mexico; AZ, Arizona; TX, Texas; MX·SLP, San Luis Potosí, Mexico; CA, California; FL, Florida; BRA, Brazil; VEN, Venezuela; MX·CHP, state of Chiapas Mexico; TRI, Trinidad; ARG, Argentina; BOL, Bolivia; PER, Peru; COL, Colombia. LCMV, the prototypic member of the lymphocytic choriomeningitis–Lassa (Old World) serocomplex, was the designated outgroup in the analyses.

Analyses of GPC sequences, N protein sequences, and nucleotide sequences included AV B1030026, 8 other viruses from North America, and 15 viruses from South America (Figure 2). Sequences in each amino acid sequence dataset were aligned by using ClustalW version 2.0.12 (*21*). The sequences in each nucleotide sequence dataset were aligned manually, and alignment was guided by the corresponding computer-generated amino acid sequence alignment. Sequence nonidentities were equivalent to uncorrected (*p*) distances.

Phylogenetic analyses of nucleotide sequences were conducted by using MRBAYES version 3.1.2 (*22*) and programs in PAUP* (*23*). Bayesian analyses used the general time reversible + proportion invariant + Γ model with a site-specific gamma distribution and the following options in MRBAYES version 3.1.2: two simultaneous runs of 4 Markov chains, 10 million generations, and sample frequency every 1,000th generation. The first 1,000 trees were discarded after review of the likelihood scores, convergence statistics, and potential scale reduction factors. A consensus tree (50% majority rule) was constructed from the remaining trees. Probability values in support of the clades were calculated a posteriori, and clades with probability values >0.95 were considered supported by the data (*20*).

## Results

Arenavirus was not isolated from any kidney samples. However, N protein gene RNA of arenavirus AV B1030022 and N protein gene RNA of arenavirus AV B1030026 were detected in samples of kidney from Mexican deer mice TK93321 and TK93325, respectively. The sequence of a 746-nt fragment of the N protein gene of AV B1030022 (GenBank accession no. JN897399) was 96.4% identical to the nucleotide sequence of the homologous region of the N protein gene of AV B1030026.

Bayesian analyses of complete GPC gene sequences (Figure 2, panel A) and complete N protein gene sequences (Figure 2, panel B) separated the Tacaribe serocomplex viruses into 4 groups (A, B, C, D). Arenavirus AV B1030026 was included in group B with Amaparí virus (AMAV), Cupixi virus (CPXV), TCRV, and the 5 viruses from South America known to cause hemorrhagic fever in humans. Group D is exclusively North American, groups A and C are exclusively South American, and probability values calculated a posteriori indicate strong support for monophyly of viruses in each group and strong support for the sister relationship between AV B1030026 and TCRV.

Nonidentities between amino acid sequences of the GPC and N protein of AV B1030026 and amino acid sequences of homologous sequences of the 8 other viruses from North America ranged from 47.8% to 52.1% and from 44.3% to 45.4%, respectively. Similarly, nonidentities between the amino acid sequences of the GPC and N protein of AV B1030026 and homologous sequences of the 8 other members of group B ranged from 24.7% to 46.8% and from 16.1% to 32.3%, respectively (Table). Nonidentity between the GPC amino acid sequences of AV B1030026 and TCRV was greater than the nonidentity between the GPC amino acid sequences of CHPV and SABV (Table), among the GPC amino acid sequences of the 5 viruses in group A (range 15.8%–23.7%), and between the GPC amino acid sequences of Latino virus (LATV) and Oliveros virus (20.6%). Last, nonidentity between the N protein amino acid sequences of AV B1030026 and TCRV was greater than the nonidentities between N protein amino acid sequences of 7 viruses from South America in 5 pairwise comparisons: AMAV and CPXV, AMAV and GTOV, CPXV and GTOV, CHPV and SABV, JUNV and MACV (Table).

**Table:** Nonidentities among amino acid sequences of glycoprotein precursors and amino acid sequences of nucleocapsid proteins of Ocozocoautla de Espinosa virus strain AV B1030026, Mexico, and 8 arenaviruses from South America*

Virus	% Amino acid sequence nonidentity								
	OCEV	AMAV	CHPV	CPXV	GTOV	JUNV	MACV	SABV	TCRV
OCEV	–	43.7	45.1	44.8	46.5	31.6	33.5	46.8	24.7
AMAV	27.7	–	44.8	30.4	29.9	42.0	42.3	46.0	45.3
CHPV	31.6	27.3	–	43.3	43.9	42.7	42.8	22.3	45.1
CXPV	27.7	17.1	28.4	–	31.2	45.2	44.8	43.5	45.7
GTOV	27.0	14.6	27.9	17.1	–	43.0	44.2	44.8	45.9
JUNV	16.1	26.3	30.3	27.1	25.2	–	29.3	44.6	32.6
MACV	17.9	25.7	29.1	25.9	23.7	12.2	–	43.6	34.9
SABV	32.3	27.7	16.2	29.3	29.5	29.9	28.3	–	47.4
TCRV	18.8	29.8	31.2	30.5	28.7	21.3	20.4	33.0	–

*Nonidentities (*p* model distances) among sequences of glycoprotein precursors are listed above the diagonal and those among sequences of nucleocapsid proteins below the diagonal. OCEV, Ocozocoautla de Espinosa virus; AMAV, Amaparí virus strain BeAn 70563; CHPV, Chaparé virus, 200001071; CPXV, Cupixi virus, BeAn 119303; GTOV, Guanarito virus, INH-95551; JUNV, Junín virus, XJ13; MACV, Machupo virus, Carvallo; SABV, Sabiá virus, SPH 114202; TCRV, Tacaribe virus, TRVL 11573.

## Discussion

Arenaviruses AV B1030022 and AV B1030026 are direct evidence that arenaviruses phylogenetically closely related to the South American hemorrhagic fever arenaviruses are enzootic in North America. Results of Bayesian analyses of GPC gene sequence data, Bayesian analyses of the N protein gene sequence data, and pairwise comparisons of amino acid sequences collectively indicate that AV B1030026 is a strain of a novel species (proposed name Ocozocoautla de Espinosa virus) in the family *Arenaviridae*, genus *Arenavirus* (*1*).

The hallmark of the arenaviruses is their ability to establish chronic infections in their respective principal hosts. The failure to isolate arenavirus from Mexican deer mice in this study could be caused by small sample size or poor specimen quality. Alternatively, a cricetid rodent other than the Mexican deer mouse is the principal host of Ocozocoautla de Espinosa virus (OCEV).

Members of the rodent family Cricetidae, subfamily Sigmodontinae (*8*) are the principal hosts of GTOV, JUNV, MACV, and other Tacaribe serocomplex viruses in South America for which natural host relationships have been well characterized (*24*). The available fossil record suggests that sigmodontine rodents originally invaded South America from North America after formation of the Panamanian Isthmus 2.5–3.5 million years ago.

The presence of OCEV in Mexico suggests that the last common ancestor of the 9 viruses in group B (Figure 2) emerged in North America. As such, arenaviruses phylogenetically closely related to OCEV, in association with cricetid rodents, may be widely distributed in North America. We note that antibody against AMAV has been found in northern pygmy mice (*Baiomys taylori*) captured in Texas and northern Mexico (*14*).

The history of the State of Chiapas includes an outbreak of a highly lethal hemorrhagic fever in 1967 (*17*). Antibody against Paraná virus (PARV) or LATV was found in convalescent-phase serum samples from persons who survived the fever, by an assay in which antibodies against PARV and LATV reacted with GTOV, JUNV, TCRV, and other Tacaribe serocomplex viruses (*25*). Of note, hemorrhagic fever in Chiapas clinically resembled hemorrhagic fevers caused by arenaviruses from South America, the outbreak was preceded by large-scale destruction of forested areas in the epidemic area, the *P. mexicanus* deer mouse is a relatively common species in forests of southern Mexico (*26*), and abundance of rodents in and around houses in the epidemic area had increased to disturbing proportions in the 3-year period before 1967 (*17*). Hypothetically, OCEV or an arenavirus phylogenetically closely related to OCEV was the etiologic agent in the hemorrhagic fever epidemic in Chiapas in 1967 and presently is the cause of a human disease that is clinically indistinct from dengue hemorrhagic fever and other severe febrile illnesses that are endemic to Chiapas.

It is generally accepted that humans usually become infected with arenaviruses by inhalation of virus in aerosolized droplets of secretions or excretions from infected rodents. Another source of infection may be ingestion of infected rodents (*27*).

Human consumption of wild rodents is common in rural areas in some regions of Mexico. For example, Mexican deer mice and other cricetid rodents are consumed by the Tzeltal Indians in the highlands of Chiapas (*28*). Future studies on arenaviruses native to North America should include work to assess whether humans who consume wild rodents or live or work in close association with cricetid rodents in the highlands of Mexico acquire illness from OCEV or other North American Tacaribe serocomplex viruses.
